# Correction of high-rate motion for photoacoustic microscopy by orthogonal cross-correlation

**DOI:** 10.1038/s41598-024-53505-2

**Published:** 2024-02-21

**Authors:** Zilong Zou, Qiuqin Mao, Renxiang Cheng, Chao Tao, Xiaojun Liu

**Affiliations:** 1 MOE Key Laboratory of Modern Acoustics, Department of Physics, Collaborative Innovation Center of Advanced Microstructures, Nanjing University, Nanjing, 210093 China; 2 School of Electronic and Information Engineering, Jinling Institute of Technology, Nanjing, 211169 China

**Keywords:** Biomedical engineering, Image processing

## Abstract

Photoacoustic imaging is a promising technology for in vivo imaging. However, its imaging performance can be hampered by motion artifacts, especially when dealing with high-rate motion. In this paper, we propose an orthogonal motion correction method that utilizes cross-correlation along orthogonal scan directions to extract accurate motion displacements from the photoacoustic data. The extracted displacements are then applied to remove artifacts and compensate for motion-induced distortions. Phantom experiments demonstrate that the proposed method can extract the motion information and the structural similarity index measurement after correction is increased by 26.5% and 11.2% compared to no correction and the previous correction method. Then the effectiveness of our method is evaluated in vivo imaging of a mouse brain. Our method shows a stable and effective performance under high-rate motion. The high accuracy of the motion correction method makes it valuable in improving the accuracy of photoacoustic imaging.

## Introduction

Photoacoustic microscopy (PAM) is an emerging biomedical imaging method, by combining optical excitation with ultra-broad bandwidth ultrasound detection^1–3^. Therefore, it has the both advantages of optical imaging and acoustic imaging^4–6^. PAM can achieve a spatial resolution in the tens of microns and provide several millimeters of imaging depths in biological tissues. These merits promise great potential for PAM in biomedical studies, such as cancer diagnostics^7^, in vivo brain imaging^8^, oral inspection^9^, coronary artery assessment^10^, and so on^26–33^.

PAM usually achieves image by point-by-point raster scanning a focused ultrasound transducer over the region of interest (ROI) to pick up photoacoustic signals generated by the pulsed laser. Data acquisition speed is limited by the repetition rate of the laser. It typically takes several minutes to complete the data acquisition procedure. For example, if the pulsed laser has a repetition rate of 1000 Hz, it requires about 60 s at least to complete a 250 × 250 point-by-point scanning. Moreover, the scan time could be much longer, for larger ROI, higher resolution, multi-wavelength illumination, or multiple data averaging.

For the application of PAM on the living body, the movement of the target due to breathing or heartbeat could alter the relative position between the PAM and the sample. Moreover, these movements could have a respiratory rate of up to 30 times per minute for human^11^ and even up to 180 times per minute for mice^12^, which is much faster than the frame rate of PAM. Eventually, this long scanning time makes PAM susceptible to artifacts due to breathing or other motions. The quick motion of the living body could significantly affect the performance of a PAM system.

Restricting the motion of living body during scanning is challenging for PAM^13–15^. Mechanical clamping and fixation methods, such as using suction or adhesive tape, have been employed to restrict the lateral movement of the region of interest. However, vertical motion along the axial direction cannot be completely eliminated due to the potential deformation of the soft coupling media between the ultrasound transducer and the sample, such as water, ultrasound gel, and polyethylene membranes. Several methods have been proposed to reduce the impact of vertical motion artifacts. A method based on detecting skin surface is introduced to correct motion by generating a smooth synthetic surface^16^. Another approach is proposed to calculate a cumulative cross-correlation surface and quantify vertical displacement to correct artifacts^17^. Retrospective respiratory gating is used to sort and cluster photoacoustic tomography images by simultaneous capturing of the animal’s respiratory waveform during photoacoustic data acquisition^18–24^. Furthermore, quasi-periodic scanning combined with a register-fusion algorithm is introduced to imaging moving targets in PAM^25^. In a word, the efficient method of motion correction is significant to improve the performance of PAM.

In this study, we propose a method to eliminate motion artifacts caused by high-rate motion. This method combines correlation along two orthogonal scan directions to correct vertical motion artifacts. Our method operates at the signal level, enabling simultaneous extraction of displacement for compensation of amplitude distortion Phantom experiments are conducted to examine the effectiveness and evaluate the performance of the proposed method. Finally, the method is verified in vivo mouse brain imaging experiments.

## Materials and methods

### PAM and the motion problem

In practice, the breathing, heartbeat, and other involuntary movements of small animals can cause vertical motion, as illustrated in Fig. 1a. This displacement will alter the position of the ultrasound transducer relative to the sample, which will result in distortion of vascular morphology and PA signal intensity.

**Figure 1:**
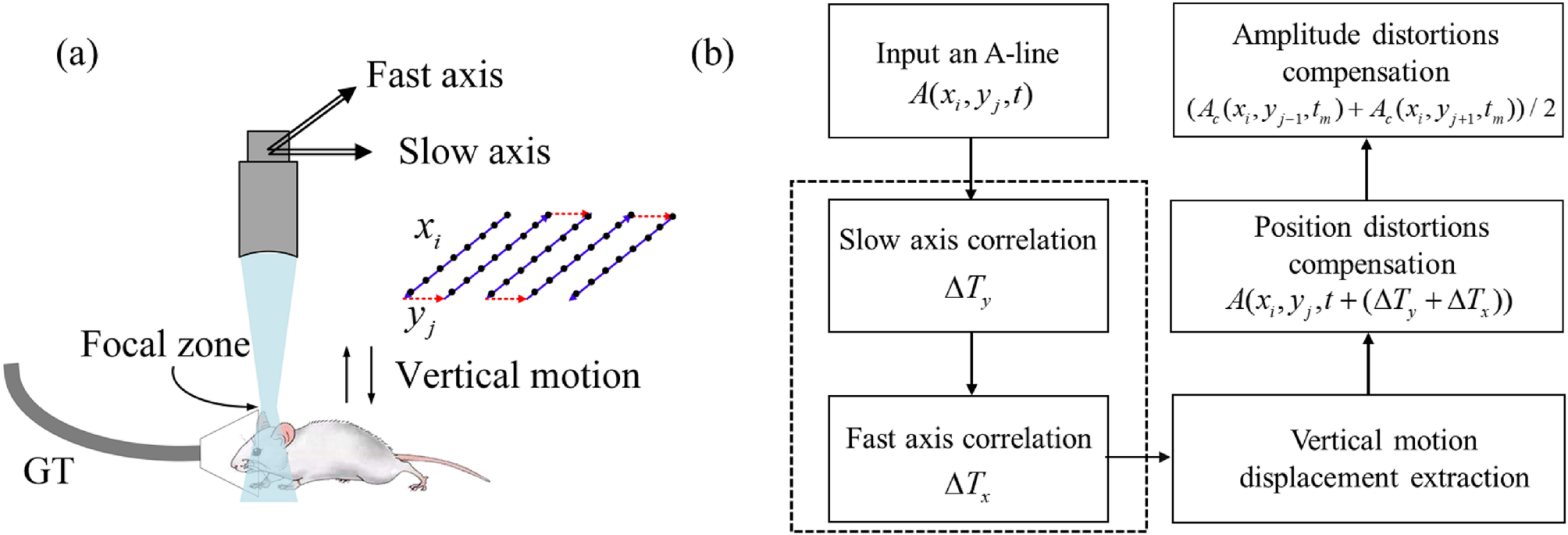
Principle of the orthogonal motion correction method. (**a**) The process of raster scanning and signal collection. GT, gas tube. (**b**) The overall flowchart of the motion correction method. The black dotted box represents the calculation of orthogonal A-lines cross-correlation.

The scanning method employed in PAM involves a fast-axis corresponds to x-axis and a slow-axis corresponds to y-axis. One-dimensional time signals (A-line) are recorded at each scan point, collecting ultrasound signal generated within the detection angular aperture of the focused detector. The transducer moves steadily at a constant speed along the x-axis to complete a B-scan. After each B-scan, the ultrasound transducer moves one raster step size along the y-axis. This process is repeated until the entire region of interest has been scanned.

Figure 1b shows the overall flowchart of our motion correction method. For each input A-line, a time shift is calculated by considering correlation to accurately quantify vertical motion. Correlation calculation is performed in two perpendicular scanning directions. Here, we name the time shift by calculating correlation along the slow axis and fast axis as Δ*T*_*y*_ and Δ*T*_*x*_, respectively. Then, we extract vertical motion displacement. Finally, we compensate for position and amplitude distortions.

### The orthogonal motion correction method

Let’s suppose the A-line received by the ultrasound transducer can be represented as *A*(*x*_*i*_, *y*_*j*_, *t*), where *t* is time, *x*_*i*_ and *y*_*j*_ are the positions of the acquisition grid in *x* and *y* directions. *i* is the coordinate in the direction between adjacent A-lines, and *j* is the coordinate in the direction between adjacent B-scans.

Since motion could change the time-of-flight for an acoustic wave from the optical absorption to the detector surface, the arrival time *T*_*c*_(*x*_*i*_, *y*_*j*_) of the corrected PA signals should be expressed as:

1 $$T_{c} (x_{i} ,y_{j} ) = T(x_{i} ,y_{j} ) + \Delta T_{y} (x_{i} ,y_{j} ) + \Delta T_{x} (x_{i} ,y_{j} )$$

Here, *T*(*x*_*i*_, *y*_*j*_) is the arrival time of the detected PA signals. The time shift Δ*T*_*y*_(*x*_*i*_, *y*_*j*_) and Δ*T*_*x*_(*x*_*i*_, *y*_*j*_) can be estimated by maximizing the cross-correlation between received signals of adjacent acquisition grid positions following the next steps.

First, the correlation along the y-axis is calculated for each A-line. The A-line at (*x*_*i*_, *y*_*j*_) is compared with the A-line at (*x*_*i*_,* y*_*j*+1_). The time shift can be estimated by maximizing the cross-correlation,

2 $$\Delta T_{y} (x_{i} ,y_{j} ) = \mathop {\arg \max }\limits_{\Delta t} [\sum\limits_{t = - \infty }^{\infty } {A(x_{i} ,y_{j} ,t + \Delta t)A(x_{i} ,y_{j + 1} ,t)} ]$$

Second, if Δ*T*_*y*_(*x*_*i*_, *y*_*j*_) ≠ 0, we calculate the correlation of the detected motion position along the x-axis. Due to the high sampling speed in x-axis, which is significantly shorter than the periods of motion, a continuous distortion emerges along the fast axis. The time shifts are accumulated, enabling accurate motion displacement estimation.

3 $$\Delta T_{x} (x_{i} ,y_{j} ) = \sum\limits_{m = 0}^{M} {\mathop {\arg \max }\limits_{\Delta t} [\sum\limits_{t = - \infty }^{\infty } {A(x_{i - m} ,y_{j} ,t + \Delta T_{y} + \Delta t)A(x_{{i - m - {1}}} ,y_{j} ,t + \Delta T_{y} )} ]}$$

The value of *M* can be estimated according to the duration of animal’s motion. A too large value could cause an over correction, while a small value will result in a less effective correction effect. The total vertical displacement can be calculated by Δ*z* = *c*(Δ*T*_*x*_ + Δ*T*_*y*_), where *c* represents the speed of sound in water. Thus, we obtain high-precision displacement results, allowing us to compensate for motion distortions effectively.

Third, the phase of the received signal is realigned based on the extracted motion displacements. The signal after correction *A*_*c*_(*x*_*i*_, *y*_*j*_, *t*) can be represented as:

4 $${\varvec{A}}_{c} (x_{i} ,y_{j} ,t) = A(x_{i} ,y_{j} ,t + (\Delta T_{y} + \Delta T_{x} ))$$

Finally, the intensity distortion caused by deviation from the focal zone is calibrated by adjacent signals. The magnitude of the signal *A*_*c*_(*x*_*i*_, *y*_*j*_, *t*_*m*_) is adjusted to the amplitude value of the adjacent signals (*A*_*c*_(*x*_*i*_,* y*_*j*-1_, *t*_*m*_) + *A*_*c*_(*x*_*i*_,* y*_*j*+1_, *t*_*m*_))/2, where *t*_*m*_ is the position of the maximum signal amplitude. For each position in the scanning plane, the above steps are repeated to achieve three-dimensional PA images. The processed image describes the motion-free PA signal.

The orthogonal motion correction method combines the correlation along orthogonal scan directions. When dealing with high-rate motion, a motion-corrupted A-line will be surrounded by A-lines that are also motion-corrupted. In this case, calculating correlation along a single scan axis is unlikely to maintain accurate estimation. To overcome these problems, correlations along orthogonal scan directions are combined to correct vertical motion artifacts. Thus, the proposed method ensures precise detection of motion artifacts and facilitates accurate motion displacement estimation.

### Experimental setup

Figure 2 depicts the schematic diagram of the PAM system used in this study. The system incorporates a Nd: YAG laser (EXPL-532-2Y, Spectra-Physics Inc., Santa Clara, USA) at a wavelength of 532 nm with a repetition rate of 10 kHz. The laser beam is coupled to a multimode fiber (MMF) using a convex lens. The output light is collimated and converted to a ring-shaped beam using a conical lens. The ring-shaped beam is then focused on the sample using an aluminum optical condenser to provide dark-field illumination^3^. A homemade spherical focused ultrasound transducer is placed in the center of the optical condenser to detect PA signals. The transducer has a central frequency of 13 MHz with a relative bandwidth of 66.7% at − 6 dB, a diameter of 8 mm, and a focal length of approximately 8 mm. The lateral resolution of the PAM system could be theoretically estimated as 0.71λ/NA = 164 μm. The detected PA signals were digitized by a data acquisition card (National Instruments, NI-5761) at a sampling frequency of 250 MHz. A 2D motorized translational stage (KSA050-11-X, ZI Corp., Beijing, China) controlled by a motion controller (MC600, ZI Corp., Beijing, China) performs x–y plane raster scanning.

**Figure 2:**
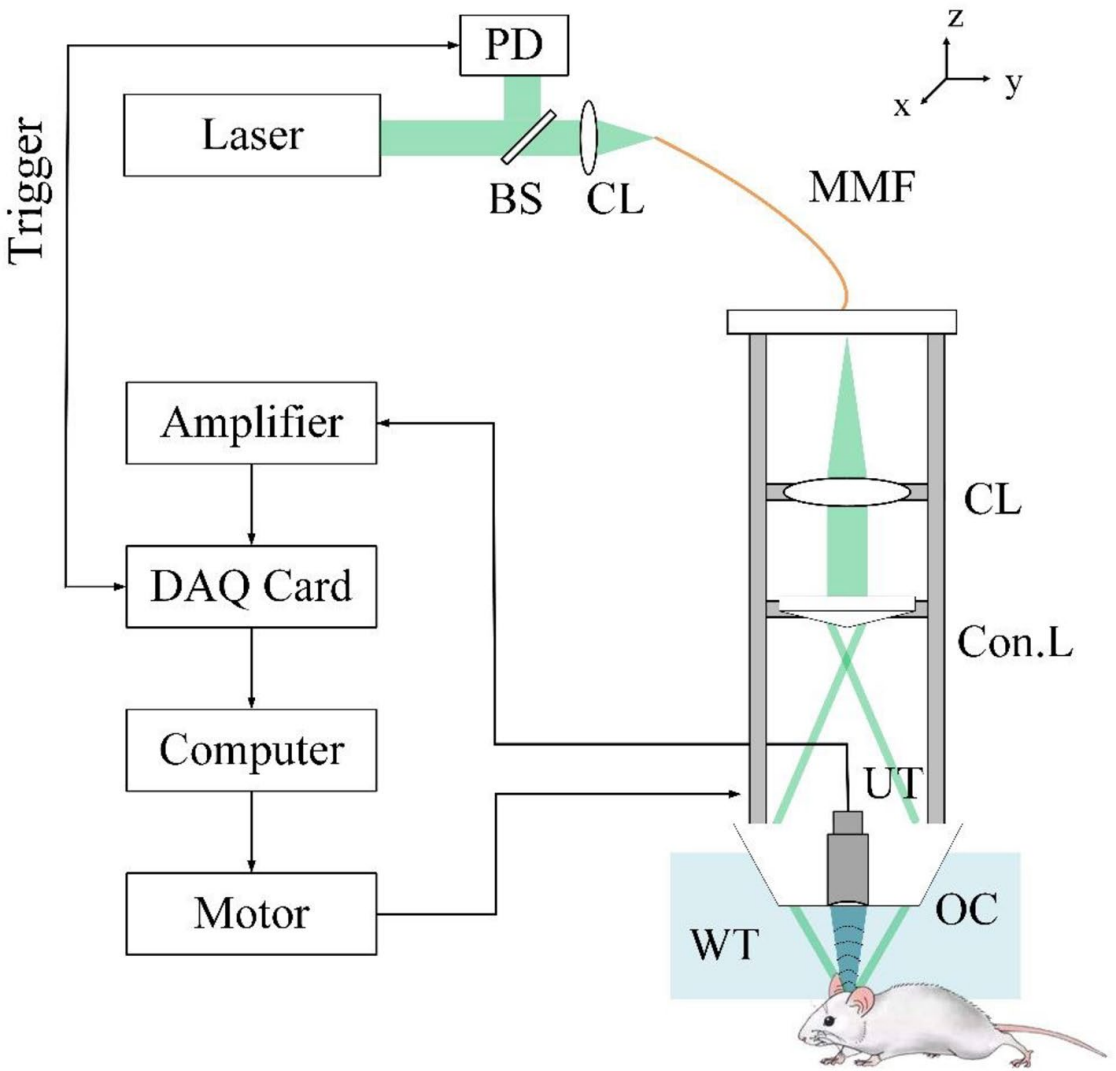
The experimental setup. *PD* photodiode, *BS* beam splitter, *CL* convex lens, *MMF* multimode fiber, *Con.L* conical lens, *DAQ Card* data acquisition card, *UT* US transducer, *OC* optical condenser, *WT* water tank.

### Animal preparation

The animal studies were performed in conformity with protocols approved by the Animal Studies Committee of Nanjing University. The study was carried out in compliance with the ARRIVE guidelines. All methods were carried out in accordance with relevant guidelines and regulations. A male nude mouse (around 6 weeks old and weighing ~ 20 g) was selected as the animal model for the in vivo imaging experiments. We initially placed the mouse in an induction box, where we anesthetized it using isoflurane gas. Once the anesthetized took effect, we moved the mouse onto the animal holder to keep it lateral fixation and utilized an animal anesthesia machine and breathing mask to maintain general anesthesia throughout the experiment. During the experiment, the concentration of anesthetic was maintained at 3%.

### Ethics declarations

The ethics approval for experiments reported in this work is provided by the Animal Studies Committee of Nanjing University.

## Results

### Motion correction in phantom experiments

A phantom experiment was conducted firstly to examine the performance of the method. The phantom used in the experiment was composed of randomly-distributed black hairs, which act as the vessels in tissue. To simulate the vertical motion of small animals, a voice coil motor was utilized to vibrate the phantom up and down. During the experiment, the voice coil motor was driven by a pulse wave signal with a duty cycle of 35% and a frequency of 3 Hz. The vertical oscillation had a maximum displacement of 0.12 mm. The scanning area was 5 × 5 mm and the scanning step size was 50 μm.

Figure 3 gives the typical waveforms of PA signals when the phantom is vertically oscillating and still. In Fig. 3a, the red line represents a motion-corrupted PA signal. The PA signal has both position and amplitude deviation. The blue dotted line in Fig. 3a depicts the reference PA signal of the still phantom at the same position, which is not corrupted by motion. Figure 3b shows the detected signal after time shift considering correlation along a single scan direction (red curves). Due to the motion-corrupted signals surround, the position distortion of the signal has been partially corrected. However, by calculating cross-correlation along orthogonal scan directions, we can get an accurate displacement. This displacement information allows us to effectively shift the distorted signal to its actual position, as illustrated in Fig. 3c. Then, we compensate for the amplitude deviation of original signal. Figure 3d presents a comparison between the signal before and after the amplitude compensation process. After compensation, the intensity distortion is rectified.

**Figure 3:**
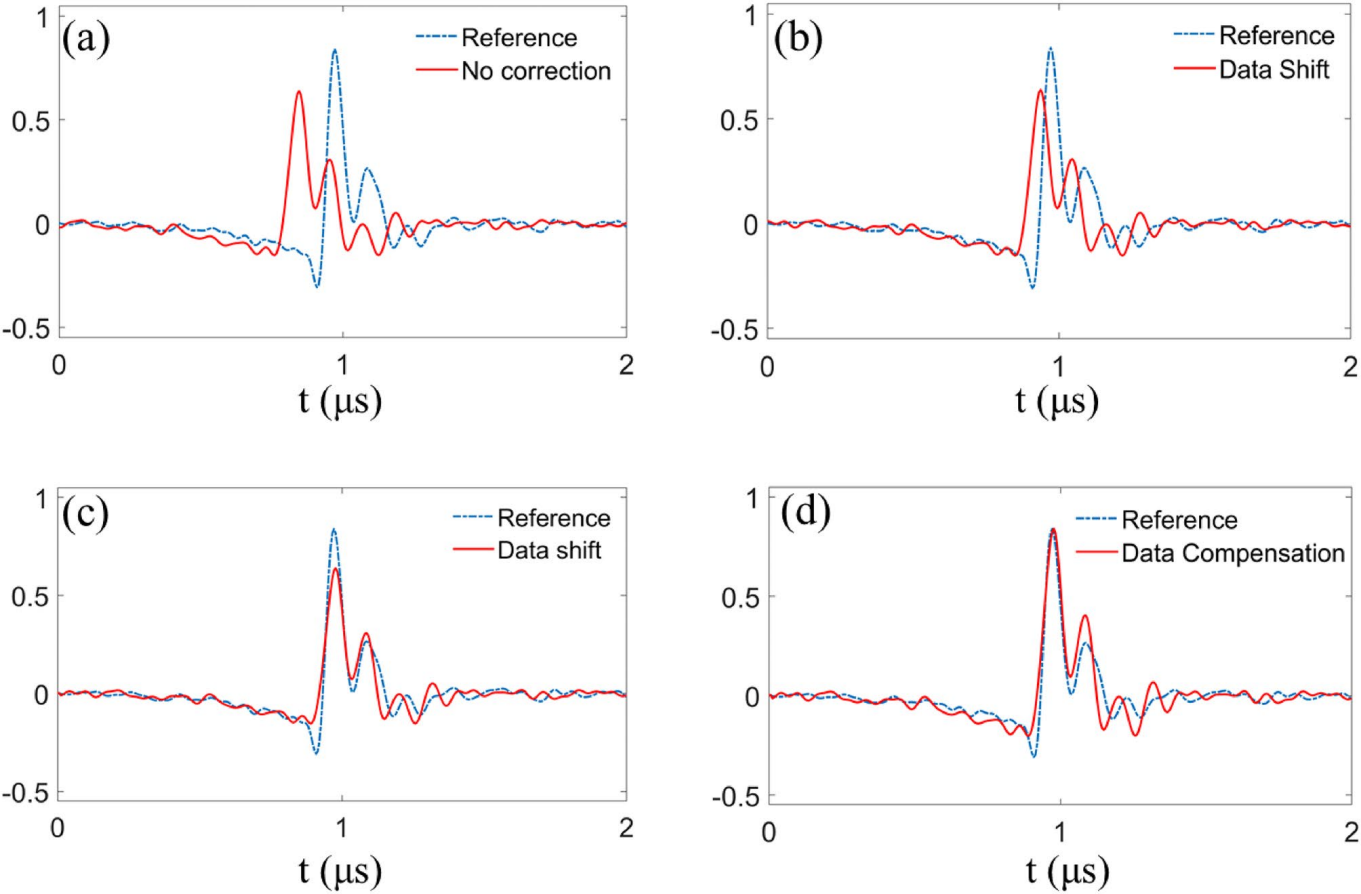
The process of orthogonal motion correction in a typical A-line. (**a**) Comparison of motion-corrupted PA signal (red curves) and the reference signal (blue dotted curves). The PA signal has both position and amplitude deviation. (**b**) The detected signal after position correction along a single scan direction (red curves) and the reference signal (blue dotted curves). (**c**) The detected signal after position correction along orthogonal scan directions (red curves) and the reference signal (blue dotted curves). (**d**) The signal processed after employing amplitude compensation (red curves) and the reference signal (blue dotted curves).

Figure 4 gives the PA imaging results of the phantom experiment. As shown in Fig. 4a, PAM provides high quality images when the phantom is still. Figure 4b gives imaging results when the phantom is vibrated vertically with a frequency of 3 Hz. Compared to the still PA image in Fig. 4a, the image in Fig. 4c contains many evident black artifacts. These artifacts result from distortion caused by motion. Due to the axial displacement, the optical absorber moves away from the focus zone, resulting in a decrease in the PA signal amplitude. Figure 4c represents the result after applying motion correction. It can be seen that the corrected image agrees well with the still PA image in Fig. 4a and the photograph in Fig. 4g. This indicates that the motion-induced artifacts are effectively reduced by the proposed motion correction.

**Figure 4:**
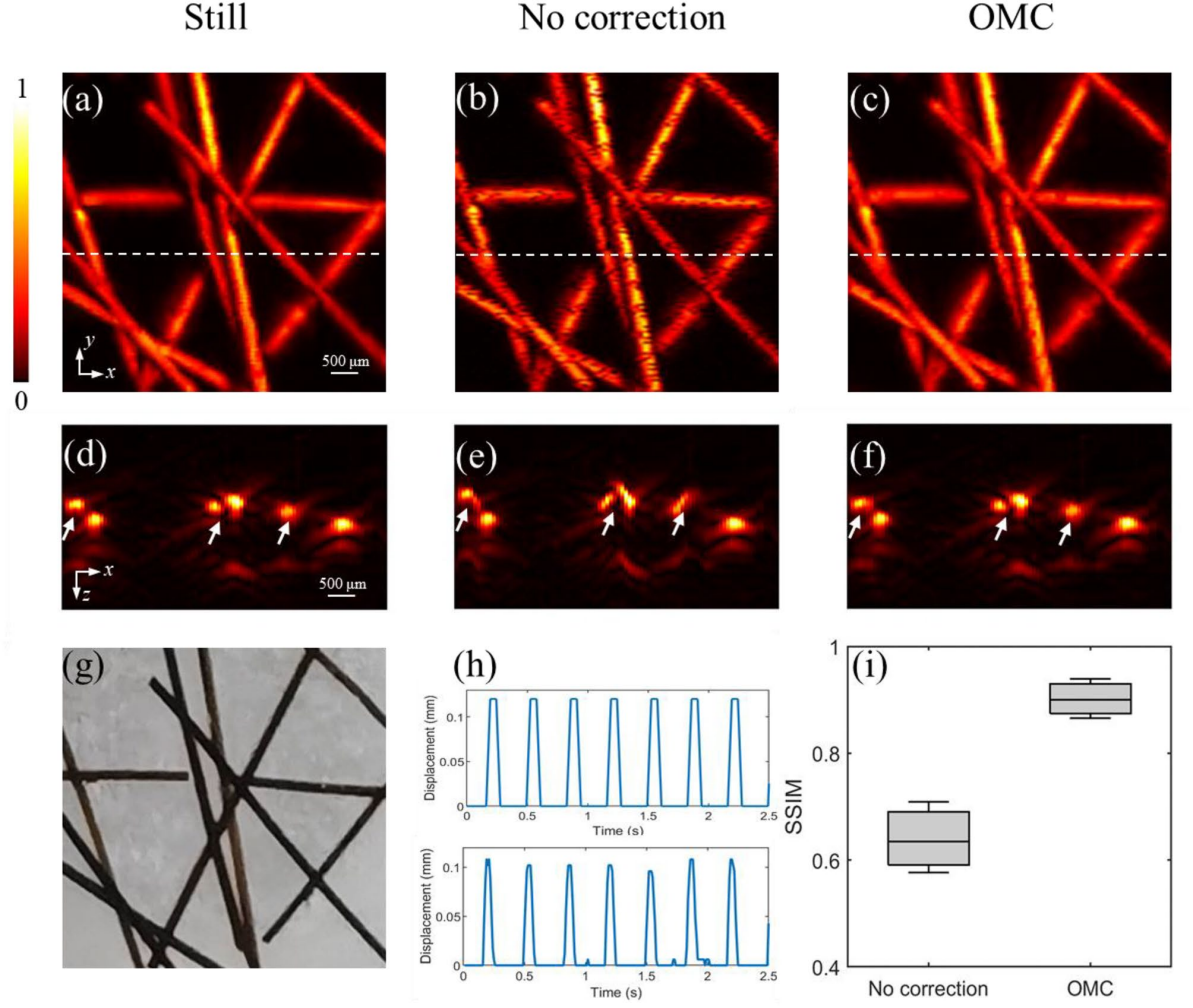
Verification of the OMC method by phantom experiments. (**a**) MAP image of the still phantom. (**b**) MAP image of the vertically oscillating phantom. (**c**) MAP image of the vertically oscillating phantom after OMC. (**d**–**f**) are the x–z MAP images along the white dashed lines in (**a**–**c**). (**g**) A photograph of the phantom. (**h**) Waveform of the vertical oscillation. Top: the actual vertical movement of the phantom. Bottom: the motion displacement extracted by OMC. (**i**) SSIM before correction and after correction.

Figure 4d–f give the images in x–z sections along the white dashed line in Fig. 4a–c. The vertical motion causes the morphological changes of the hairs, as shown in Fig. 4e. However, after correction, the hairs in Fig. 4f exhibit their original shape. The corrected image is almost the same as the still image in Fig. 4d. The deformed images of hairs are corrected. Figure 4h plots the waveform of the vertical vibration. The top represents the input waveform and the bottom represents the retrieve waveform. The agreement between the waveforms demonstrates that the proposed method can accurately estimate the vertical motion. Figure 4i quantifies the effect of motion correction through the structural similarity index measurement (SSIM)^34^. The SSIM analysis is performed on the PA images in x–z cross-sections, comparing the PA images of vertically moving samples with the still sample. The calculation is conducted on each B-scan along the y-axis. A larger SSIM value means that the corrected image is more similar to the still image, indicating a good correction performance. As shown in Fig. 4i, orthogonal motion correction (OMC) has a larger SSIM value and much more modest fluctuations. After correction, the mean SSIM value increases from 0.635 to 0.899, which is significantly higher than before correction. The OMC method successfully corrects motion artifacts and improves image quality.

### Motion correction at different frequencies

Subsequently, we compared the performance of different methods under various degrees of living animal motion. In this experiment, the phantom vibrated at different frequencies of 1Hz, 3Hz, and 5Hz, but maintained the same maximum displacement amplitude of 0.12mm. The scanning area was 5 × 5 mm, with a scanning step size of 50 μm.

Figure 5a,d,g give the MAP imaging results of 1Hz, 3Hz and 5Hz, respectively. Compared to Fig. 5a, Fig. 5d,g exhibit more black line artifacts. For the same scanning speed and scanning time, the increased motion frequency will result in more black line artifacts on the MAP image. Figure 5b,e,h illustrate the results after single-axis motion correction (SMC). In the case of 1Hz, SMC can effectively correct the artifacts. However, as the frequency increases, the correction effect of SMC deteriorates. Many artifacts persist in the processed images, as shown in Fig. 5e,h. This is because SMC is unable to effectively correct adjacent corrupted A-lines. The increasing frequency will lead to more artifacts in similar positions. The correlation between adjacent corrupted A-lines remains high. Consequently, it is difficult for SMC to detect and address these artifacts. However, after OMC, it can hardly find artifacts in PA images, as shown in Fig. 5c,f,i. The OMC method successfully corrects adjacent corrupted A-lines and shows a better performance compared to SMC. This is because the OMC method combines correlation along orthogonal scan directions, extracting a more accurate motion displacement estimation.

**Figure 5:**
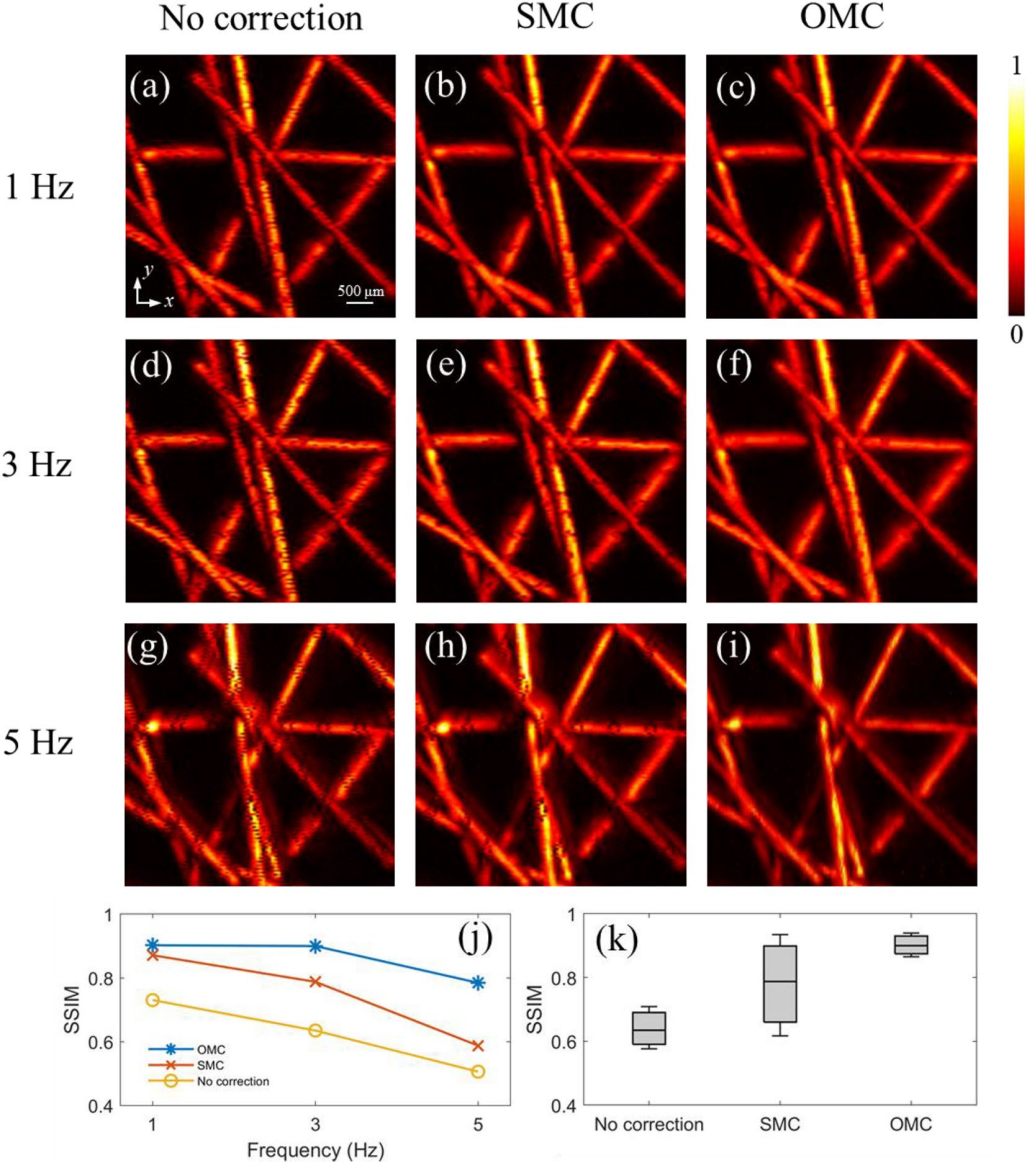
Comparison of motion correction performance under motion frequencies 1Hz, 3 Hz, and 5 Hz. (**a**–**c**) MAP images of the vertically oscillating phantom at 1 Hz. (**d**–**f**) MAP images of the vertically oscillating phantom at 3 Hz. (**g**–**i**) MAP images of the vertically oscillating phantom at 5 Hz. (**j**) The mean SSIM without processed, processed by SMC and OMC at different frequencies. (**k**) The statistical analysis of SSIM value at 3 Hz.

We also conducted SSIM analysis to quantify the correction effects under various motion degrees, as illustrated in Fig. 5j. The mean SSIM value of the no correction images (yellow line) decreases because the increasing frequency results in more artifacts. The SSIM value of the SMC (red line) also decreases sharply, suggesting a deteriorating correction effect due to increased artifacts in similar positions. However, the SSIM of OMC (blue line) remains high, showing excellent correction performance. The average SSIM values of different methods are presented in Table 1. To further illustrate the stability and performance of the OMC method, Fig. 5k presents a comparative analysis between the SSIM values with no correction, SMC, and OMC at 3Hz. The mean structural similarity index measurement value and standard deviation of no correction, SMC, and OMC are 0.635 ± 0.074, 0.788 ± 0.170, and 0.899 ± 0.034. The mean SSIM value after OMC increased by 26.5% and 11.2% compared to no correction and SMC. The OMC method has significantly more modest fluctuations in SSIM values, presenting successful correction of adjacent corrupted A-lines. This demonstrates the effectiveness of OMC in correcting motion artifacts, even in the presence of high-rate motion.

**Table 1:** The average SSIM values for different method.

Frequency	1Hz	3 Hz	5Hz
No correction	0.730	0.635	0.506
SMC	0.871	0.788	0.588
OMC	0.902	0.899	0.785

### Motion correction in vivo experiments

To validate the practicability of the proposed orthogonal motion correction method, we applied the method to correct in vivo image of the mouse brain. The scanning range of mouse brain was 12 mm × 10 mm with a step size of 30 μm.

Figure 6 gives the imaging results of the mouse brain. Fig. 6a is the original photoacoustic MAP image, where the depths are encoded by the color. Fig. 6b is the depth encoded MAP image of the mouse brain after correction. For comparison, Fig. 6c is a photograph of the mouse brain after trimming the scalp. Due to the axial displacement caused by the movement of the mouse, the optical absorption deviates from the focal zone, resulting in signal attenuation. As seen in the MAP image in Fig. 6a, there are many evident black line artifacts. To better present, Fig. 6d–f present the 3D rendering display of the mouse brain. The PAM image in Fig. 6d is the original image without any motion correction. As shown, the motion of animals induces serious imaging distortions and changes the shape of the blood vessels in the image. Fig. 6e gives the image processed by SMC. Although this approach corrects some distortions, there are still many artifacts remaining in the image. The high-rate motion of mice leads to artifacts in similar positions. SMC is unable to detect and address these artifacts because the adjacent corrupted A-lines maintain a high correlation value. Fig. 6f gives the PAM image processed by the proposed OMC method. All motion artifacts have been effectively removed. The vascular structures in the brain appear smooth and the image quality is significantly improved.

**Figure 6:**
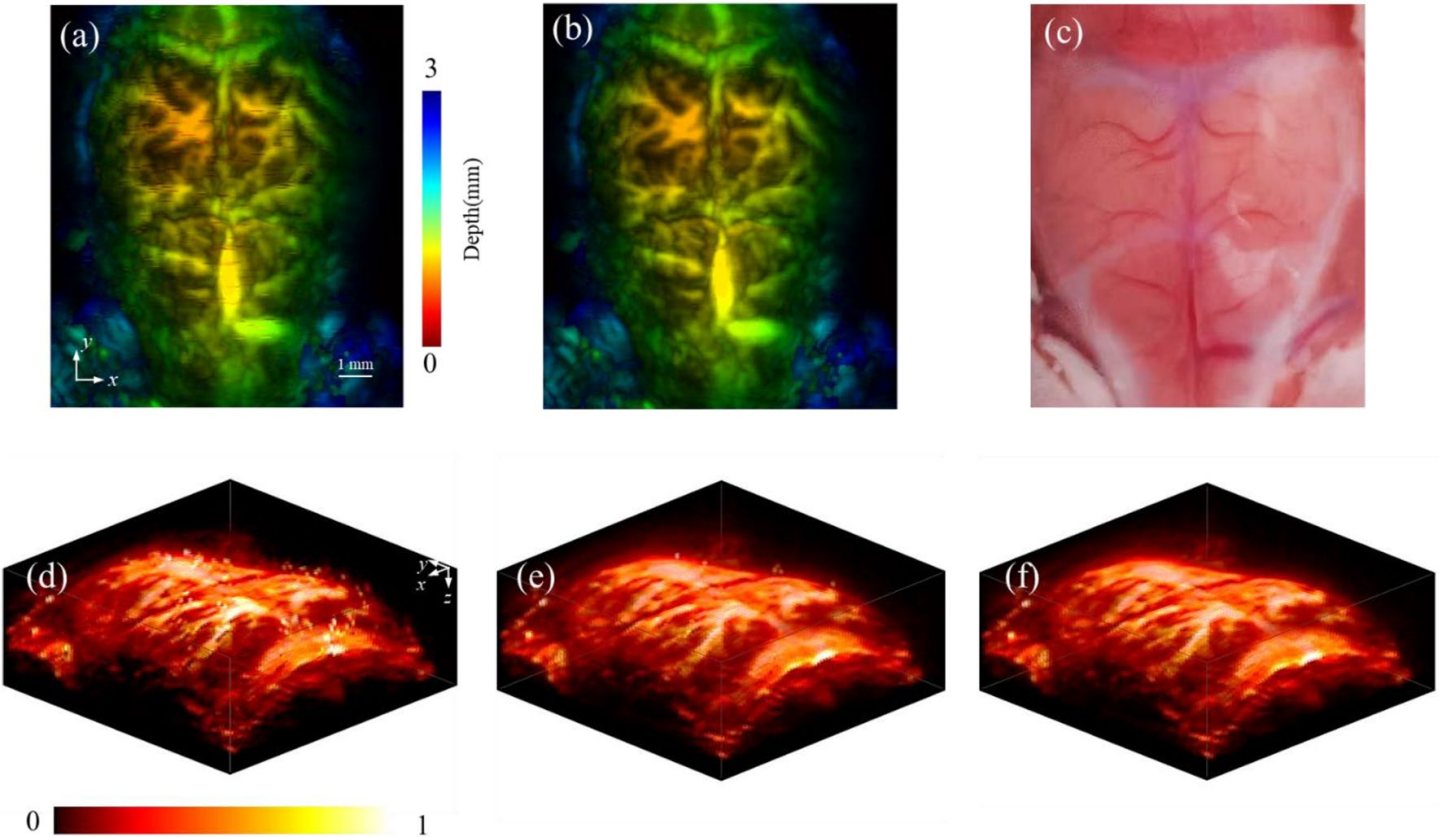
Application of the motion correction method on the in vivo mouse brain imaging. (**a**) The depth encoded MAP image of the mouse brain without motion correction. (**b**) The depth encoded MAP image of the mouse brain after processing by OMC. (**c**) A photograph of the mouse brain. (**d**) Volume rendering without motion correction. (**e**) Volume rendering after processing by SMC. (**f**) Volume rendering after processing by OMC.

Figure 7 shows the brain images from x-z and y-z cross-sections. Figure 7a,d are the images without any motion correction. In the x-z section, the fast-scanning speed along the x-axis results in continuous morphologic distortion, as shown in Fig. 7a. Conversely, the slow-scanning speed along the y-axis direction leads to spiking distortion in the y-z cross-section, as shown in Fig. 7d. Figure 7b,e show the images corrected by the SMC method. Some artifacts persist in the images because the high-rate motion of mice leads to deterioration in the performance of the SMC method. Figure 7c,f give images obtained by the OMC method. To illustrate the enhancement further, Fig. 7g–i features the cross-sectional images enlarged in the white dashed boxes in Fig. 7d–f. The minor displacement is still shown in Fig. 7h because of the imperfect correction. However, by implementing OMC, we can get a more accurate displacement in Fig. 7i. The OMC method effectively corrects all motion distortions and significantly improves image quality.

**Figure 7:**
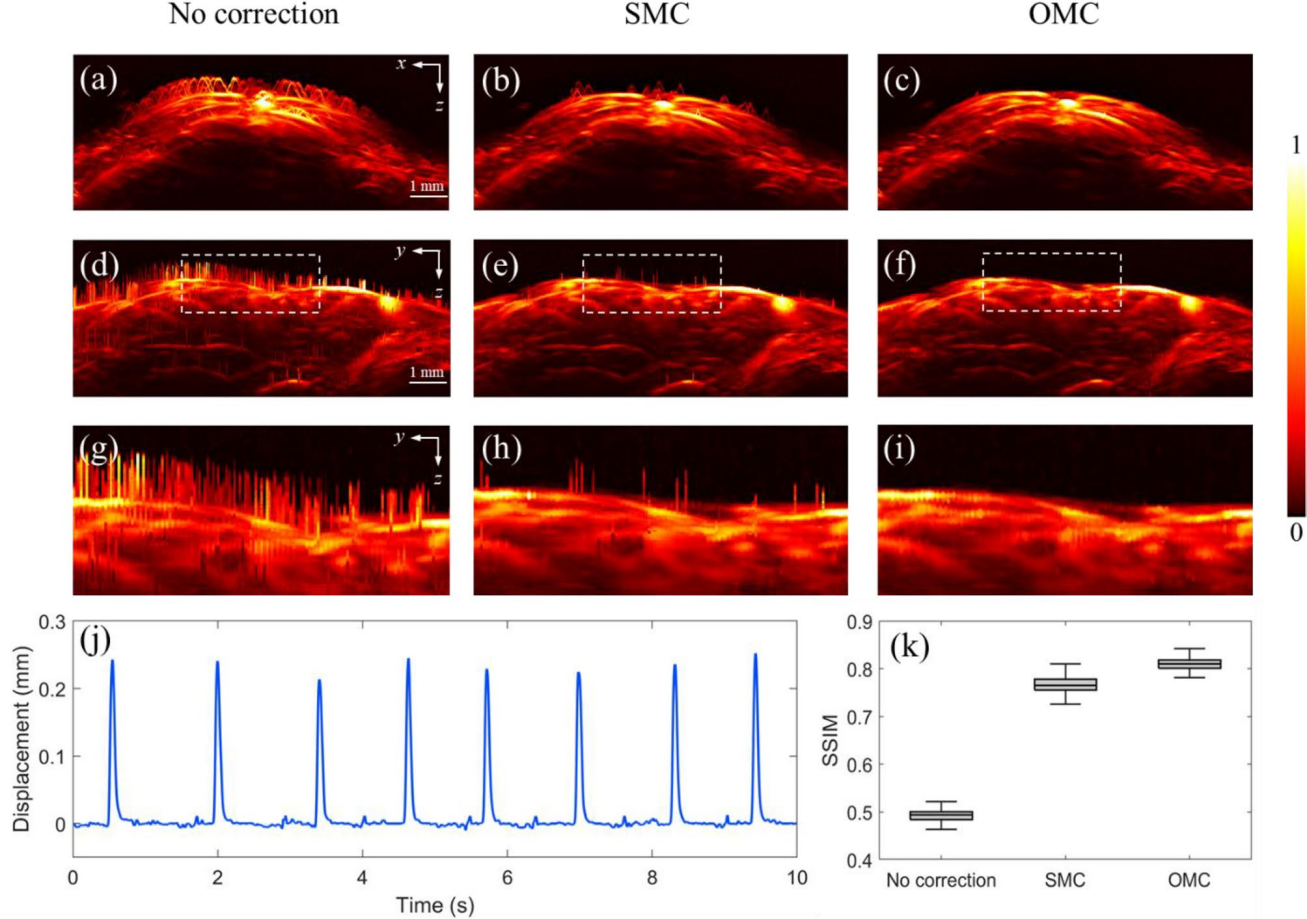
Comparisons of SMC and OMC. (**a**–**c**) The x–z MAP images with no correction, SMC and OMC. (**d**–**f**) The y–z MAP images with no correction, SMC and OMC. (**g**–**i**) The enlarged details in the white dashed boxes in (**d**–**f**). (**j**) The waveform of motion displacement extracted by OMC. (**k**) SSIM before correction, after processing by SMC and OMC.

As shown in Fig. 7j, we capture a typical motion period. The displacement waveform extracted by our method matches well with the respiratory waveform of mice reported in other research^12^. We also quantify the effect of motion correction in vivo imaging by conducting SSIM analysis on images in x-z cross-sections along y-axis, as illustrated in Fig. 7k. The mean structural similarity index measurement value and standard deviation of no correction, SMC, and OMC are 0.494 ± 0.030, 0.765 ± 0.039, and 0.810 ± 0.028. Images corrected by the OMC have modest fluctuations and higher SSIM values. Our orthogonal motion correction method eliminates motion displacement completely, improving the quality of PA images.

## Discussion

In this study, we proposed an orthogonal motion correction method to extract motion information and remove artifacts. Compared to the existing method, the orthogonal motion correction method combines cross-correlation along orthogonal scan directions and demonstrates better performance in correcting motion artifacts and improving image quality. It shows high accuracy and robustness, particularly in imaging the high-rate moving sample. Another major advantage of our method is that it only relies on data acquisition without the need for external sensors or gating. Most previous motion correction methods employ respiration gating, which introduces additional devices and synchronization challenges. By combining our method, a set of PA data is sufficient for monitoring motion and achieving complete motion correction. Although our method has some advantages, it has its limitations. Our method can correct well with vertical motion but has limited correction ability to correct artifacts caused by horizontal motion. The method can be further improved by considering correlation along the z-axis. Additionally, we focus on addressing these pulse-like high-frequency motion with relatively small displacements in this study. The correction of larger displacements can be discussed in future work. The experiment results demonstrate the effectiveness and stability of our method for motion correction in PAM, suggesting its promising applications in clinical settings. Furthermore, our method can be evaluated for more in vivo applications, imaging regions with high-rate motion such as the belly or the back of the animals, where the motion patterns are complex, including breathing, heartbeat, and other involuntary movements. By achieving robust motion correction under high-frequency movement, our method has the potential to improve photoacoustic imaging and enable simultaneous physiological monitoring in clinical applications.

## Data Availability

The datasets used and/or analyzed during the current study are available from the corresponding author (C.T. or X.L.) upon reasonable request.
